# Supplementary material for: Fungal mycetoma and pregnancy: An association with costly and difficult management, about a case
Source: PLoS Negl Trop Dis. 2023 Nov 13;17(11):e0011372. doi: 10.1371/journal.pntd.0011372 (PMC10688872; doi:10.1371/journal.pntd.0011372)
Supplement: Attachment — Submitted filename: PNTD-D-23-00553.docx [file pntd.0011372.s001.docx]

PNTD-D-23-00553

Mycetomas are defined as all pathological process in which fungal or actinomycosis Please revise the etiology of actinomycetoma. There are several bacteria causing this disease.

The patient returned to the clinic 2 months later with a progressive pregnancy, which led to the discontinuation of itraconazole and terbinafine, which were replaced by amoxicillin-clavulanic acid 2g/day. – Why did you stop antifungal therapy and what is the reason to give antibiotics to your patient?

The patient had to spend 10,687 euros to achieve this result, divided between the purchase of drugs, the costs of surgery, hospitalization costs and payment of complementary examinations without counting travel costs between Louga and Dakar.

Why do you changed itraconazole to voriconazole? Is there any difference of in vitro sensibility between this two triazole? The therapy expenses is a social political problem in Senega and this is not a scientific issue.

How do you identified the etiologic agent from your patient?
