# Supplementary material for: Fungal mycetoma and pregnancy: An association with costly and difficult management, about a case
Source: PLoS Negl Trop Dis. 2023 Nov 13;17(11):e0011372. doi: 10.1371/journal.pntd.0011372 (PMC10688872; doi:10.1371/journal.pntd.0011372)
Supplement: Attachment — Submitted filename: Response to Reviewers.docx [file pntd.0011372.s002.docx]

Answers to reviewers' comments

PNTD-D-23-00553

🡺Mycetomas are defined as all pathological process in which fungal or actinomycosis please revise the etiology of actinomycetoma. There are several bacteria causing this disease.

Answer: I have given the definition of mycetomas, adopted at the 1st International Symposium on Mycetoma in Venezuela in 1978.

🡺The patient returned to the clinic 2 months later with a progressive pregnancy, which led to the discontinuation of itraconazole and terbinafine, which were replaced by amoxicillin-clavulanic acid 2g/day. – Why did you stop antifungal therapy and what is the reason to give antibiotics to your patient?

Answer: We had stopped the itraconazole because of its possible side effects on pregnancy, and introduced antibiotics because the lesions were superinfected. However, bacteriological analyses were not carried out to find the causal germ. It's the wording of the sentence that's not right, so we're going to correct it.

🡺The patient had to spend 10,687 euros to achieve this result, divided between the purchase of drugs, the costs of surgery, hospitalization costs and payment of complementary examinations without counting travel costs between Louga and Dakar.

*Why do you changed itraconazole to voriconazole? Is there any difference of in vitro sensibility between this two triazole?

Answer:

*The therapy expenses is a social political problem in Senegal and this is not a scientific issue.

Answer: this aspect is included to demonstrate the high cost and the difficulty of managing fungal mycetomas in underdeveloped countries like Senegal especially if associated with pregnancy.

🡺How do you identified the etiologic agent from your patient?

Answer:

The diagnosis was based on the black color of the grain, the culture on sabouraud-chloramphenicol medium and the histopathological presentation of the grain

------------------------------------------O------------------------------------------

Description of the changes we have made in the manuscript

Introduction:

🡺Line 4: Classified since 2016……

🡺Line 7:…Cases have been reported in the literature concerning the occurrence of mycetomas in pregnancy…

🡺Line 9:…some of which were progressing well…

Observation:

🡺Line 16: Treatment with amoxicillin/clavulanic acid 2g/day was initiated in view of the inflammatory appearance of the lesions, which were suspected of superinfection

🡺Line 25: C - reactive protein

Discussion:

🡺Line 3: Although reported by several authors this is not a very common occurrence

🡺Line 10: and al.

🡺Line 12: these authors believe

🡺Line 6: In many countries, the high cost of management of neglected tropical skin diseases is linked to the high cost of drugs
