# Supplementary material for: Fungal mycetoma and pregnancy: An association with costly and difficult management, about a case
Source: PLoS Negl Trop Dis. 2023 Nov 13;17(11):e0011372. doi: 10.1371/journal.pntd.0011372 (PMC10688872; doi:10.1371/journal.pntd.0011372)
Supplement: Attachment — Submitted filename: Response letter.docx [file pntd.0011372.s003.docx]

Response letter

To,

The editorial board,

PLOS Neglected Tropical Diseases,

Dear editors,

Subject: Submission of revised manuscript number PNTD-D-23-00553R1

Thank you for your email dated of 14 August 2023, enclosing the reviewers’ comments. We have carefully reviewed the comments and have revised the manuscript accordingly. Our responses are given in a point-by-point manner below. Changes to the manuscript are shown in underline/yellow.

We hope the revised version is now suitable for publication and look forward to hearing from you in due course.

Sincerely,

Niare NDOUR, MD

Department of Dermatology, Hospital Institute of Social Hygiene, Dakar, Senegal

- Response to Reviewer 1:

Thank you for your review of our paper. We have answered each of your points below.

🡺This is a single case
-Was the *M. mycetomatis* identified by culture or by molecular methods?

*Response:

*M. mycetomais* was identified by histopathological examination and by mycological culture

-Can you be more specific about the surgery ie was this visually complete excision. What doses of voriconazole were used and when was the patient changed to itraconazole

*Response:

-The surgery was visually complete

-The doses of variconazole was: 200 mg × 2/day

- Itraconazole was stopped 2 months later when patient returned to the clinic with a progressive pregnancy. It was stopped because of its possible side effects on pregnancy.

🡺In Table 1 what does thrust mean ? Worsening ??"

*Response:

Thrust means a worsening

🡺The patient had to develop several previous pregnancies on this site of mycetoma, the outcome of which was favourable". Does this mean that the patient had been pregnant several times before in the presence of the same mycetoma but had not developed any complications ? If so this isn’t what is said in the discussion where it is implied that the patients’ mycetoma was aggravated in all pregnancies
*Respose:

This means that most of these pregnancies progressed well, i.e. there was a normal delivery with a healthy newborn. However, there was a worsening of the mycetoma.
🡺Chain Reactive Protein –

*Response:

Thank you for the comment

We have corrected:

C-reactiveProtein

🡺"Only a few cases have been reported in the literature concerning this association". I would be more careful here as you haven’t carried out a full literature review and there are more cases. So this is a real and significant association. I would change this to ….Cases have been reported in the literature concerning this association. You again say this is a rare occurrence in the discussion – I would rephrase this to say Although reported by several authors this is not a very common occurrence.

*Reponse:

Thank you,

We have corrected

🡺This author believes - there are 9 authors!

*Response:

Thank you,

We have corrected by “these authors believe”

🡺Voriconazole, first injected. Presumably this was by standard iv infusion rather than direct infiltration of the lesion

*Response:

This was by iv infusion rather

🡺Table 2
Is there a missing drug in the first column ? Also some of the data is repeated

Thank you for comment,

Yes, itraconazole was missing in the first column, we have corected

🡺Conclusion. You might point out that this drug cost problem is a very real one in many countries where the best management of Skin NTDs is compromised by the high cost of medications - in this case antifungals

*Response:

Thank you,

Thank you, we have taken your comment into account: “In many countries, the high cost of management of neglected tropical skin diseases is linked to the high cost of drugs”

- Response to Reviewer 2:

Thank you for your review of our paper. We have answered each of your points below
🡺The microbiologic identification of *M. mycetomatis* is not included

*Response:

*M. mycetomais* was identified by histopathological examination and by mycological culture. We have added this aspect
🡺The authors do not explained the reason to stop antifungal therapy and changing to antibiotics

*Response:
We had stopped the itraconazole because of its possible side effects on pregnancy, and introduced antibiotics because the lesions were superinfected. However, bacteriological analyses were not carried out to find the causal germ. It's the wording of the sentence that's not right, so we have corrected it by: “treatment with amoxicillin/clavulanic acid 2g/day was initiated in view of the inflammatory appearance of the lesions, which were suspected of superinfection”
🡺Table 1 is not necessary and should be substituted by a time lining

*Response:

Table 3 shows the characteristics of the different associations between mycetoma and pregnancy.
🡺Table 3 is not necessary

*Response:
Thank you,

We've deleted it
🡺The authors should focus their discussion in the clinical and radiological outcome and especially the use of Antifungal drugs during pregnancy.
In my opinion, advocacy related to therapy costs is non relevant.

The authors should change the focus of their manuscript.
They should discuss the antingungal drugs during pregnancy
The costs of antifungals and or the therapy in African countries is not scientifically interessante, but they could resume this issues in a single sentence

*Response:

Thank you for comment,

We wanted to show the high cost by estimating the overall cost of treating a case of mycetoma in a tropical area. Since the actual cost is not often reported in studies
