# Supplementary material for: Fungal mycetoma and pregnancy: An association with costly and difficult management, about a case
Source: PLoS Negl Trop Dis. 2023 Nov 13;17(11):e0011372. doi: 10.1371/journal.pntd.0011372 (PMC10688872; doi:10.1371/journal.pntd.0011372)
Supplement: Attachment — Submitted filename: Response letter.docx [file pntd.0011372.s005.docx]

Response letter

To,

The editorial board,

PLOS Neglected Tropical Diseases,

Dear editors,

Subject: Submission of revised manuscript number PNTD-D-23-00553R1

Thank you for your email dated of 24 September 2023, enclosing the reviewers’ comments. We have carefully reviewed the comments and have revised the manuscript accordingly. Our responses are given in a point-by-point manner below. Changes to the manuscript are shown in underline/yellow.

We hope the revised version is now suitable for publication and look forward to hearing from you in due course.

Sincerely,

Niare NDOUR, MD

Department of Dermatology, Hospital Institute of Social Hygiene, Dakar, Senegal

- Response to Reviewer 1:

Thank you for your review of our paper. We have answered each of your points below.

🡺The authors provided answers to my queries such as in Table 1 Thrust. This does mean Worsening - so it is important to change Thrust to Worsening in the table.

*Response:

Thank you for the comment

We have corrected: we have replaced thrust by worsening

🡺 I would also change exeresis to surgery

*Response:

Thank you fr the comment,

We have replaced exerosis by surgery

- Response to Reviewer 2: No recommendations
